# Elevated Oestrogen Receptor Splice Variant *ERαΔ5* Expression in Tumour-adjacent Hormone-responsive Tissue

**DOI:** 10.3390/ijerph7113871

**Published:** 2010-11-02

**Authors:** Siân E. Taylor, Imran I. Patel, Paras B. Singh, Caroline M. Nicholson, Helen F. Stringfellow, R. K. Gopala Krishna, Shyam S. Matanhelia, Pierre L. Martin-Hirsch, Francis L. Martin

**Affiliations:** 1 Centre for Biophotonics, Lancaster Environment Centre, Lancaster University, Bailrigg, Lancaster LA1 4YQ, UK; E-Mails: siantaylor@gmail.com (S.E.T.); masterped786@googlemail.com (I.I.P.); parassingh@btinternet.com (P.B.S.); martin.hirsch@mac.com (P.L.M.H); 2 Lancashire Teaching Hospitals NHS Trust, Sharoe Green Lane, Fulwood, Preston PR2 9HT, UK; E-Mails: caroline.nicholson@lthtr.nhs.uk (C.M.N.); helen.stringfellow@lthtr.nhs.uk (H.F.S.); shyam.matanhelia@lthtr.nhs.uk (S.S.M.); 3 Wockhardt Hospitals & Kidney Institute, 111A, Rash Behari Avenue, Kolkata 700029, India; E-Mail: rkgkrishna@yahoo.com (R.K.G.K.)

**Keywords:** endometrial cancer, oestrogen receptor, prostate cancer, real-time RT PCR, splice variant

## Abstract

Susceptibility to prostate or endometrial cancer is linked with obesity, a state of oestrogen excess. Oestrogen receptor (ER) splice variants may be responsible for the tissue-level of ER activity. Such micro-environmental regulation may modulate cancer initiation and/or progression mechanisms. Real-time reverse transcriptase (RT) polymerase chain reaction (PCR) was used to quantitatively assess the levels of four ER splice variants (*ERαΔ3*, *ERαΔ5*, *ERβ2* and *ERβ5*), plus the full-length parent isoforms *ERα* and *ERβ1*, in high-risk [tumour-adjacent prostate (*n* = 10) or endometrial cancer (*n* = 9)] *vs*. low-risk [benign prostate (*n* = 12) or endometrium (*n* = 9)], as well as a comparison of UK (*n* = 12) *vs*. Indian (*n* = 15) benign prostate. All three tissue groups expressed the ER splice variants at similar levels, apart from *ERαΔ5*. This splice variant was markedly raised in all of the tumour-adjacent prostate samples compared to benign tissues. Immunofluorescence analysis for ERβ2 in prostate tissue demonstrated that such splice variants are present in comparable, if not greater, amounts as the parent full-length isoform. This small pilot study demonstrates the ubiquitous nature of ER splice variants in these tissue sites and suggests that ERαΔ5 may be involved in progression of prostate adenocarcinoma.

## 1. Introduction

Prostate and endometrial cancers are the most common cancers of the reproductive tract in UK-resident men and women, respectively. Indeed, prostate cancer (CaP) is the most common cancer overall and the second most common cause of cancer death in men (Office for National Statistics 2007 data). Both exhibit a marked geographical variation in incidence, with endometrial cancer and clinically-significant CaP being far more common in affluent countries, such as the USA and Western Europe [1]. Even within individual nations, variation exists between urban and rural populations [2,3]. Both cancers have been linked with a high saturated fat intake in addition to other dietary and/or environmental factors but obesity plays a major role [4,5].

Tissues of the reproductive organs are highly influenced by oestrogens. The endometrium proliferates in response to oestrogenic stimulation; when unopposed by progesterone, this commonly results in hyperplasia and occasionally, in endometrioid endometrial cancer [6]. It has recently been suggested that oestrogen also plays a role in prostatic carcinogenesis [7], possibly via *TMPRSS2:ERG* fusion transcripts [8]. The *CYP19*-encoded aromatase, responsible for the peripheral conversion of adrenal and testicular androgens to oestrogens, is highly expressed in adipose tissue. This is the primary source of circulating oestrogen in men and postmenopausal women [9]. The UK and India have clear differences in diet and average body mass index. Therefore differing oestrogen status may contribute to the marked dissimilarity in the prevalence of CaP between these populations. Reproductive differences between richer and poorer countries, especially in contraceptive use and childbearing, are also important contributors to the incidence of endometrial cancer.

Tissues of the reproductive organs display complex regulation of sex steroid secretion. Feedback loops exist with the pituitary and hypothalamus to control circulating gonadal hormone levels. These have less influence on peripherally-generated oestrogens. Oestrogen is a powerful promoter of cell division and so it is plausible that a further level of regulation occurs in sensitive tissues at the cellular level. There is compelling circumstantial evidence to suggest that this occurs, at least in part, via oestrogen receptor (ER) splice variants [10]. ERs exist as two separately encoded isoforms, ER alpha (*ERα*) and ER beta (*ERβ1*). Alternative processing of precursor mRNA results in a range of splice variant forms, several of which are translated into proteins. Most ERα splice variants involve exon deletions; ERαΔ3 lacks a DNA binding domain but is otherwise intact and ERαΔ5 is truncated, missing the entire ligand binding domain. Both influence the activity of full-length ERα. ERαΔ3 inhibits ERα-mediated transcription but also activates the vascular endothelial growth factor promoter [11]. ERαΔ5 binds DNA (with weak constitutive activity) and competitively inhibits the binding of ERα [12]. ERβ2 and ERβ5 both have an alternative exon 8. This alters their ability to bind ligand and recruit cofactors [13]. Both are widely expressed, often at similar levels to the full-length ERβ1 [14]. ERβ2 does not bind ligand or directly stimulate transcription. ERβ2 and ERβ5 are able to form dimers with ERα and ERβ1. ERβ5 preferentially binds ERα, inhibiting its effect [13,15].

The four splice variants selected for this study (ERαΔ3, ERαΔ5, ERβ2 and ERβ5) have all been previously found in uterine tissue [16–18]. No ERα splice variants have previously been described in the prostate but both ERβ2 and ERβ5 are known to be present [19]. This small pilot study aimed to discover whether the level of expression of these ER splice variants is linked to the risk of malignant transformation. In order to do this we studied three pairs of hormone-sensitive tissues: firstly, normal endometrium *vs.* tumour-adjacent (TA) tissue; secondly, benign prostate *vs.* TA tissue; and, thirdly, benign prostate tissues from high-risk (UK) *vs.* low-risk (Indian) populations. Our objective was to determine whether a putative role for ER splice variants in the pathogenesis of prostate and endometrial cancers could be identified; this would then need to be verified in a larger cohort study.

## 2. Materials and Methods

### 2.1. Study Participants

This study was conducted with appropriate ethical approval at two centres; for UK-resident participants under LREC nos. 06/Q1309/76 and 05/Q1302/83 (Preston, Chorley and South Ribble Ethical Committee), whilst for India-resident participants institutional ethical approval in Workhardt hospital (Kolkata, India) was obtained.

For benign prostate tissues (PROS), patients undergoing trans-urethral resection of the prostate (TURP) were identified and prospectively consented based on their having a low risk of harbouring CaP (no previous history of CaP, benign-feeling gland on digital rectal examination and prostate-specific antigen (PSA) < 10 ng/mL serum); except for one patient, PROS 9, who had an open prostatectomy for a >200 g-sized prostate (PSA = 34 ng/mL, final histology benign). For TA prostate tissues, appropriate patients undergoing retro-pubic radical prostatectomy (RRP) for biopsy-identified localized CaP were identified and prospectively consented. Among those undergoing RPP for localized CaP, study participants were chosen with low volume of the disease on prostate biopsies and low PSA (<15 ng/mL). All PROS and TA prostate tissues were collected in Preston (UK) from Caucasian British, UK-resident men. The Indian prostate specimens (IND) were collected in Kolkata (India) from India-resident, Indian men undergoing TURP.

For endometrial tissues, appropriate women were identified and prospectively consented. TA endometrial tissues were obtained from patients with biopsy-proven endometrial cancer undergoing hysterectomy as part of their initial treatment. In order to minimise variation only tissues from women with grade 2 endometrioid endometrial carcinoma were used in this study. Control tissues were obtained from patients undergoing hysterectomy for benign conditions. All were pre-menopausal women in the proliferative phase of a natural menstrual cycle.

### 2.2. Tissue Collection and Storage

Following surgical resection, prostate chips were immersed in a cold 0.9% saline solution; other tissues were placed in a dry, clean plastic pot. All specimens were transported directly to the laboratory. Tissues were dissected by a consultant histopathologist under standard clean conditions. For endometrial tissue, the cavity was exposed by first amputating the cervix and, then opening the anterior wall of the uterus. In benign cases, a small sample of representative endometrium (≈5 mm × 5 mm) was shaved off the underlying myometrium. In malignant cases, the tumour was macroscopically identified and a piece of normal-looking endometrium sampled as above, on the opposite side of the uterus from the tumour site (precise distances dependent on size of uterus, usually 2–3 cm from tumour edge). For prostate TA tissue, macroscopically-normal material assumed to be CaP-free was selected. This comprised material from the lobe that showed no or minimal CaP on pre-operative biopsy. A piece of tissue measuring approximately 1.5 cm × 0.3 cm was incised from the most peripheral and posterolateral aspect of the gland. Gross tumour is easily identified in macroscopic uterine and prostate specimens, although using this method it is not possible to exclude small areas of premalignant disease or early carcinoma. Benign prostate tissue obtained from TURP did not require dissection. Specimens were placed in RNAlater solution (QIAGEN Ltd., UK), kept at 4 °C for 24 h and then transferred for storage at −85 °C for gene expression analysis. Time between surgical resection and placement in RNAlater or formalin was <15 min. The tissues from India were transported to the UK with adequate precautions taken to maintain the appropriate temperature throughout the journey.

### 2.3. Quantitative Real-time Reverse Transcriptase Polymerase Chain Reaction (RT-PCR)

The method of RNA extraction, reverse transcription and real-time RT-PCR for prostate and endometrial tissues has been described previously [20,21]. Briefly, tissue was ground under liquid nitrogen. Total RNA extraction was performed using the Qiagen RNeasy® Kit in combination with the Qiagen RNase-free DNase kit (QIAGEN Ltd.). RNA (0.4 μg) was reverse transcribed in a final volume of 20 μL containing Taqman® reverse transcription reagents (Applied Biosystems, UK): 1 × Taqman RT buffer; MgCl_2_ (5.5 mM); oligo d(T)16 (2.5 μM); dNTP mix (dGTP, dCTP, dATP and dTTP; each at a concentration of 500 μM); RNase inhibitor (0.4 U/μL); reverse transcriptase (MultiScribe^™^) (1.25 U/μL) and RNase-free water. Reaction mixtures were then incubated at 25 °C (10 min), 48 °C (30 min) and 95 °C (5 min). cDNA samples were stored at −20 °C prior to use.

Primers (Table 1) for *ERα*, *ERβ* and the endogenous control *β-ACTIN* were chosen using Primer Express software 2.0 (Applied Biosystems) and designed so that one primer spanned an exon boundary. Specificity was confirmed using the NCBI BLAST search tool. The splice variant primers *ERαΔ3, ERαΔ5, ERβ2* and *ERβ5* were designed and specificity confirmed using the Primer-BLAST tool on the NCBI website. One of the pair was designed across the splice boundary, with at least six bases overlapping, to ensure maximum specificity. All primers (Table 1) were validated. Quantitative real-time PCR was performed using the ABI Prism 7000 Sequence Detection System (Applied Biosystems). Reaction mixtures contained 1 × SYBR® Green PCR master mix (Applied Biosystems); forward and reverse primers (Invitrogen, Paisley, UK) at a concentration of 300 nM; a 20 ng cDNA template; made to a total volume of 25 μL with sterile H_2_O. Thermal cycling parameters included activation at 95 °C (10 min) followed by 60 cycles each of denaturation at 95 °C (15 sec) and annealing/extending at 60 °C (1 min). Each reaction was performed in triplicate and “no-template” controls were included in each experiment. Dissociation curves were run to eliminate non-specific amplification, including primer-dimers.

### 2.4. Immunofluorescence

Tissues were fixed in formalin prior to wax-embedding and subsequent immunofluorescence staining of tissue sections (4-μm thick) was performed manually. Staining took place following de-waxing and re-hydration. High-temperature antigen retrieval was performed by heating the tissue sections in citrate buffer (pH 6) or glycine/EDTA (pH 8) for 3 min, under full pressure in an electric pressure cooker. Sections were then permeabilised using 0.1% Triton X-100 for 20 min, after which they were rinsed in PBS buffer (pH 7.4). Endogenous avidin/biotin was blocked using a streptavidin/biotin blocking kit (Vector Labs, UK), then washed in PBS twice for 2 min, followed by incubation in 5% normal goat serum in PBS (GSPBS) for 20 min. Anti-ERβ (ab288) and anti-ERβ2 (MCA2279ST) antibodies were purchased from Abcam and AbD serotec, respectively. Antisera dilutions were 1:50 (anti-ERβ) and 1:50 (anti-ERβ2) in GSPBS. Tissue sections were incubated with primary antisera overnight at 4 °C in a moist chamber. For each immunolabelling, negative controls in which the primary antibody step was replaced by non-specific antibody were run. Tissue sections were washed three times in PBS for 5 min, followed by three 5-min washes in distilled H_2_O. Slides were incubated with secondary biotin-conjugated goat anti-mouse IgG antisera (1:200; Vector Labs, UK) in GSPBS for 30 min, followed by two washes in PBS for 5 min. Tissue sections were then incubated in tertiary streptavidin fluorescein (1:100; Vector Labs) in PBS for 15 min, after which they were washed twice for 5 min each with PBS. After the final wash, coverslips were mounted using vector shield (Vector Labs) containing propidium iodide (PI). Immunofluorescence images were acquired using a Leica TCS SP2 confocal system (Leica Microsystems, Germany), equipped with a DMIRE2 microscope, × 40 objective lens (NA 1.25) and 488 nm argon laser line. Detection was acquired *via* two internal photomultiplier tubes (PMT) over the range 500–540 nm for fluorescein (green— to localise antibody-labelled protein) and 624–707 nm for PI (red— to stain nuclei). Control sections were used to identify tissue auto-fluorescence and non-specific staining. Subtraction was carried out by decreasing fluorescein PMT voltage until all auto-fluorescence, either non-specific- and/or majority of lipofuscin-derived, was removed from the negative control images. These parameters were saved to the system and applied to all the subsequent test slides to identify and localise specific antibody staining. Images were processed using Leica confocal software (version 2.61).

## 3. Results

### 3.1. Proliferative Endometrium vs. Grade 2 Endometrioid TA Tissue

The range of averaged threshold cycle (C_T_) values of amplified cDNA for *ERα* in benign endometrium were 23.9–29.0 and, in TA tissue 23.0–27.2. For *ERαΔ3*, the range was 28.6–32.5 in benign endometrium and 28.3–32.2 in TA tissue. For *ERαΔ5*, the range was 30.2–33.7 for benign endometrium and 30.0–33.3 for TA tissue. For *ERβ1*, the range was 20.7–36.1 for benign endometrium and 24.7–37.2 for TA tissue. For *ERβ2*, the range was 32.3–34.5 for benign endometrium and 30.9–33.4 for TA tissue. Finally, the range for *ERβ5* was 35.1–58.7 for benign endometrium and 33.9–38.9 for TA tissue. Therefore, all of the tissue samples examined expressed full-length *ERα* and *ERβ1* together with all four splice variants. Overall, transcripts for *ERα* and its splice variants were present at higher levels than *ERβ* and its splice variants (see Supplementary Information, Table 1S). There was a trend towards greater relative expression of *ERβ5* in TA tissue compared with benign endometrium (Table 2). This was not conclusive, however, and the origin of the tissue did not appear to influence the levels of the other splice variants or full-length ERs.

### 3.2. Benign Prostate vs. Tumour-adjacent Prostate

The range of averaged C_T_ values of amplified cDNA for *ERα* in benign prostate were 31.6–41.3 and in TA tissue 28.8–37.7. For *ERαΔ3*, the range was 32.9–48.2 in benign prostate and 33.5–40.1 in TA tissue. For *ERαΔ5*, the range was 39.1–56.1 for benign prostate and 34.2–48.1 for TA tissue. For *ERβ1*, the range was 31.2–37.2 for benign prostate and 32.6–36.5 for TA tissue. For *ERβ2*, the range was 31.7–37.2 for benign prostate and 23.3–33.5 for TA tissue. Finally, the range for *ERβ5* was 37.5–57.1 for benign prostate and 36.4–8.2 for TA tissue (see Supplementary Information, Table 2S). Expression of full-length *ERα* and *ERβ1* were detected in all benign and TA prostate tissues. All four splice variants were detected, although *ERβ5* was expressed at low levels and not demonstrable in several samples (PROS 1, PROS 2 and PROS 7; Table 3).

**Figure 1:**
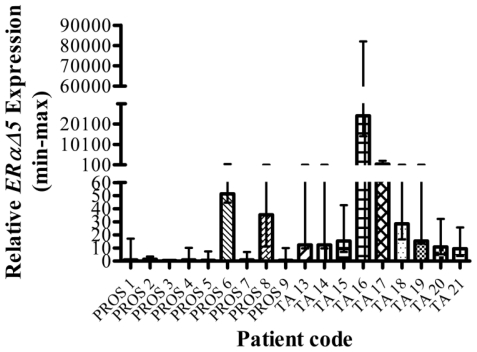
Graphical representation of relative *ERαΔ5* expression (bars) with min-max levels (lines) in normal (PROS) *vs.* tumour-adjacent (TA) prostate tissue. The y-axis is split-scale to allow all data to be plotted together.

*ERαΔ5* expression was detected at higher levels in TA tissue than benign tissue, with raised expression in 2 of 9 benign tissues and 9 of 9 TA tissues. The difference between the highest and the lowest expression levels was large. For *ERαΔ5* in TA tissue, the range was 9.5–24,154.4 and for normal tissue, the range was 0.6–51.4; such a marked difference in the expression profile of this ER splice variant indicates a significant underlying role in maintaining the adjacent cancer and would justify further investigation (Figure 1). Additionally, these tissues were validated by a single Pathologist with >25 y experience; although one could not absolutely guarantee that they were free of focal CaP, one would expect that the vast majority would be. In light of this observation, these results suggest a significant role for *ERαΔ5* in prostate TA tissue. It is interesting that two of the benign tissues exhibited higher levels of this mRNA transcript; this could be due to either *ERαΔ5* being diagnostic of future disease or unidentified CaP. One TA tissue (TA 16) had very high levels of expression of *ERα* (300.3) and both of its splice variants (*ERαΔ3*-250.2, *ERαΔ5*-24,154.4). This tissue came from a 65-y-old man with a PSA level of 6.5 and a low Gleason grade of 3 + 3 (see Supplementary Information, Tables 4S, 5S). After excluding this tissue, the mean expression level of *ERαΔ5* in TA tissue was 63.3, as opposed to 10.3 in benign prostate. Finally, the expression levels of both the ERs and the four splice variants studied varies greatly between tissue samples. This was particularly notable for *ERβ1* and *ERβ2* in addition to *ERαΔ5* (described above). Some prostate tissues (*e.g.*, PROS 8, PROS 2, TA 19) had high relative expression of both ERs and several splice variants (Table 3).

### 3.3. UK vs. India Benign Prostate

The range of averaged C_T_ values of amplified cDNA for *ERα* in UK prostate were 30.2–39.2 and in Indian prostate were 33.7–36.8. For *ERαΔ3*, the range was 32.8–40.2 in UK prostate and 35.9–39.1 in Indian prostate. For *ERαΔ5*, the range was 38.0–45.4 for UK prostate and 38.3–42.4 for Indian prostate. For *ERβ1*, the range was 30.8–39.8 for UK prostate and 32.1 ND for Indian prostate. For *ERβ2*, the range was 33.2–50.0 for UK prostate and 33.4–53.9 for Indian prostate. Finally, the range for *ERβ5* was 36.7–51.4 for UK prostate and 37.0–55.1 for Indian prostate (see Supplementary Information, Table 3S).

All tissues described here were benign and obtained from TURP. *ERα* and *ERβ1* were expressed in all tissues bar one, IND 5, which lacked *ERβ1. ERβ2* and *ERβ5* were expressed in all samples tested, as was *ERαΔ3*, but *ERαΔ5* was not detected in the sample IND 2. Overall there was no detectable difference between the UK and the Indian prostate tissue in expression levels of either the full length *ERα* or *ERβ1* or any of the splice variants (Table 4).

**Figure 2:**
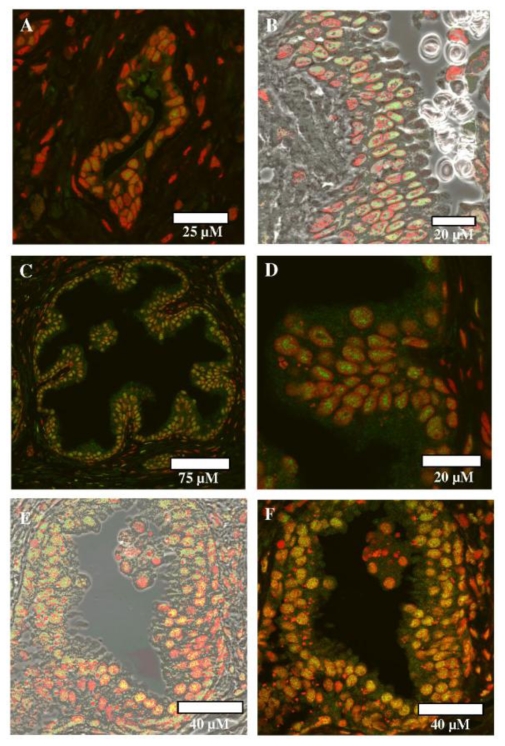
Representative photomicrographs in human prostate of staining by immunofluorescence over the range 500–540 nm for fluorescein (green antibody label identifying the spatial location of the protein) and 624–707 nm for PI (red-stained nuclei). (A) ERβ in benign prostate tissue (PROS 12); (B) ERβ in benign prostate tissue (PROS 12); (C) ERβ2 in benign prostate tissue (PROS 12); (D) ERβ2 in benign prostate tissue (PROS 12); (E) ERβ2 in benign prostate tissue (IND 8); and, (F) ERβ2 in benign prostate tissue (IND 8).

Figure 2 shows the fluorescent immunolabelling of ERβ1 in benign prostate (2A, 2B) compared to that for its splice variant ERβ2 in UK benign prostate tissue (2C, 2D) and India benign prostate tissue (2E, 2F). As expected, a primarily nuclear–associated staining pattern is noted with ERβ1 (Figure 2A), and this is clearly shown when the fluorescein (antibody, *i.e.*, green) and PI (nuclear, *i.e.*, red) positive staining is superimposed on a phase contrast background (Figure 2B). In UK benign prostate tissue at low- (Figure 2C) and high-power (Figure 2D), clear nuclear-associated staining for the splice variant ERβ2 is observed. An equally high level of staining for ERβ2 in India benign prostate tissue, which is again clearly nuclear-associated, was observed (Figure 2E, 2F). Of note, in the small number of examples examined in this study the staining for ERβ2 appeared to be more intense than that associated with its full-length parent isoform. By imposing the fluorescent images on a phase contrast background (Figure 2B, 2E), one better visualises the spatial location of the protein (labelled green) with regards to the cell nuclei (labelled red) within the cells. Of course, the more important splice variant to investigate would be ERαΔ5; however, to the best of our knowledge the anti-ERαΔ5 antibody is not currently available.

## 4. Discussion

TA tissue is useful in that it has undergone the same environmental exposure and has the same genetic source as a cancer arising elsewhere in the organ but it lacks the chaotic deregulation associated with malignancy. It is therefore valuable in assessing the status of the tissue prior to carcinogenesis. We hypothesized that altered levels of ER splice variants, perhaps secondary to an underlying abnormal oestrogen balance, are present in these TA or high-risk tissues and, so potentially are involved in the malignant process.

A large difference was found in the TA *vs.* normal prostate tissue in the level of *ERαΔ5*, with increased expression in all 9 of the RRP samples but only 2/9 of the benign TURP samples. The mean expression level in TA tissue was also over six-times higher than that in normal tissue (63.3 *vs.* 10.3), even after excluding an outlying value. ERαΔ5 is a truncated receptor and lacks most of the ligand-binding domain. It has constitutive activity, but only 5% of that of the full-length receptor and competitively inhibits the activity of ER*α* by blocking DNA-binding sites. Although the role of ERα has not been fully established in the prostate, it is frequently involved in growth promotion. The function of ERα may be more complex in the prostate as it is unlikely that inhibiting this activity could promote CaP. It is possible that this raised level of ERαΔ5 is a contributory factor in preventing the TA tissue from undergoing malignant transformation.

The explanation for this variation may not be a difference between TA and benign tissue but be due to a difference in the prostate tissue sampled; for example, different operations may favour tissue from slightly different zones. As ERαΔ5 is present at low levels, there are wide confidence intervals and this is a small sample; it is plausible that this difference is due to chance alone. In addition, the expression level of *ERαΔ5*, whilst increased, is still much lower than the expression level of *ERα*, and may be too low to have any inhibitory effect. No other splice variant demonstrated any difference between the two groups. It is likely that ERαΔ3, ERβ2 and ERβ5 are not involved in any field effect in the early stages of premalignant transformation in prostate tissue. All of the RRPs were performed after biopsy-detected malignancy; however, two were found to contain benign tissue only. The tumours in the other seven were of Gleason grade 6 to 8, with the majority (5/9) being grade 6. Recent work has discovered that increased nuclear ERβ2 and ERβ5 in CaP are associated with a poor prognosis [19]. It is possible that higher-grade CaPs would have higher levels of ERβ2 and ERβ5 in adjacent tissue, although TA15, the only Gleason grade 4 + 4 TA tissue, did not have significantly increased levels of these splice variants. The ER splice variant expression levels for the UK and Indian prostate tissues were comparable. A previous study has found similar levels of gene expression of phase I/II metabolising enzymes between a UK and Indian cohort, but clear differences were found on immunohistochemistry [21]. It would be interesting to discover whether this is also the case with the ER splice variants.

In endometrial tissue none of the splice variants tested differed between TA tissue and normal benign tissue. Previous work has found that ERβ5 is raised and that the number of ERα splice variants is increased in endometrial carcinoma [18,22]. This does not appear to be the case in TA tissue. If environmental or endogenous oestrogens do influence these processes, it is probably not via altered expression of these ER splice variants. This study has several limitations. The splice variants examined are present in small quantities and a highly sensitive technique, such as real-time RT PCR, is needed to quantify them. Confidence intervals are sometimes wide as the concentrations are occasionally at the limits of detection. We have used 60 cycles of RT-PCR as several of these splice variants are present at very low levels. This increases the risk of non-specific amplification and results were only counted if at least 2 of the 3 triplicate wells had similar results. Also, it was not possible to obtain a complete dataset for the prostate tissues due to limited cDNA. Cases and controls were not age or otherwise matched and, as this was planned as a pilot study, the number of tissues studied is not large.

Despite these limitations, the study demonstrates that prostate tissue does normally contain ERα splice variants at quantifiable levels; this has not previously been described at this tissue location. At physiological concentrations, it is known that oestrogens can induce genetic damage [23], so understanding the mechanisms by which they act is fundamentally important to understanding carcinogenic processes in these target tissues [24–26]. This is the first study of ER splice variants in TA endometrial or prostate tissue and, the first of ER splice variants in normal tissue in populations at differing risk of developing CaP (*i.e.*, UK *vs.* India). Whilst the results are predominantly negative, the findings related to ERαΔ5 in TA *vs.* benign prostate are potentially important and are worthy of more extensive study.

## Figures and Tables

**Table 1:** Primer sequences used for RT-PCR.

Gene Symbol	Primer	Sequence (5′–3′)
*ERα*	*ERα*-F	TGG ACA GGA ACC AGG GAA AAT
	*ERα*-R	GAG ATG ATG TAG CCA GCA GCA T

*ERαΔ3*	*ERαΔ3*-F	AGA AGT ATT CAA GGG ATA CGA AAA G
	*ERαΔ3*-R	ATC ATC TCT CTG GCG CTT GT

*ERαΔ5*	*ERαΔ5*-F	AGG GTG CCA GGA ACC A
	*ERαΔ5*-R	GAT GTA GCC AGC AGC ATG TC

*ERβ1*	*ERβ*-F	TGT AAA CAG AGA GAC ACT GAA AAG GAA
	*ERβ*-R	CCT CTT TGA ACC TGG ACC AGT AA

*ERβ2*	*ERβ2*-F	GCA TGC GAG GGC AGA A
	*ERβ2*-R	TTC TTT AGG CCA CCG AGT TG

*ERβ5*	*ERβ5*-F	CAC GGA GGG TGA AGT GAT TT
	*ERβ5*-R	ATT CCA AAT GAG GCA TTC ATT

*β-ACTIN*	*β-ACTIN*-F	CCT GGC ACC CAG CAC AAT
	*β-ACTIN*-R	GCC GAT CCA CAC GGA GTA CT

F, forward primer; R, reverse primer.

**Table 2:** Benign proliferative endometrium (N) *vs.* grade 2 endometrioid tumour-adjacent (TA) endometrial tissue: relative gene expression (min-max expression levels).

Patient code	*ERα*	*ERαΔ3*	*ERαΔ5*	*ERβ1*	*ERβ2*	*ERβ5*
N 1	1 (c)* (0.69–1.46)	1 (c) (0.71–1.41)	1 (c) (0.71–1.42)	1 (c) (0.71–1.41)	1 (c) (0.64–1.57)	1 (c) (0.50–2.02)
N 2	1.06 (0.92–1.23)	1.54 (1.32–1.81)	0.83 (0.72–0.96)	1.95 (1.28–2.96)	1.53 (0.95–2.46)	3.41 (2.58–4.51)
N 3	2.29 (1.68–3.13)	4.31 (3.11–5.97)	2.24 (1.71–2.93)	0.90 (0.52–1.58)	1.08 (0.65–1.81)	1.15 (0.62–2.16)
TA 1	1.07 (0.77–1.49)	1.02 (0.78–1.33)	0.55 (0.40–0.75)	7.36 (4.75–11.41)	1.47 (0.99–2.19)	8.90 (6.53–12.13)
TA 2	1.43 (1.29–1.58)	1.77 (1.54–2.02)	0.73 (0.65–0.83)	1.44 (1.01–2.05)	2.82 (1.88–4.24)	2.64 (1.61–4.33)
TA 3	1.12 (0.88–1.43)	1.03 (0.82–1.29)	0.74 (0.52–1.04)	0.98 (0.79–1.22)	1.77 (1.34–2.33)	1.00 (0.59–1.69)

N 4	1 (c) (0.91–1.10)	1 (c) (0.88–1.14)	1 (c) (0.88–1.14)	1 (c) (0.96–1.05)	1 (c) (0.70–1.43)	1 (c) (0.74–1.34)
N 5	0.61 (0.49–0.76)	1.42 (1.08–1.87)	0.85 (0.59–1.23)	2.32 (2.10–2.57)	1.82 (1.32–2.49)	2.06 (0.43–9.80)
N 6	0.93 (0.72–1.20)	0.87 (0.53–1.42)	1.14 (0.85–1.54)	0.05 (0.03–0.06)	0.62 (0.31–1.27)	2.56 (1.36–4.82)
TA 4	2.13 (1.91–2.38)	1.77 (1.62–1.94)	2.49 (2.19–2.82)	0.02 (0.01–0.03)	0.70 (0.38–1.31)	16.19 (10.38–25.23)
TA 5	2.88 (2.56–3.25)	2.13 (1.89–2.40)	2.24 (1.88–2.65)	0.01 (0.01–0.02)	0.57 (0.43–0.76)	2.92 (1.28–4.28)
TA 6	2.16 (1.88–2.48)	1.73 (1.49–2.02)	1.11 (0.86–1.44)	0.03 (0.02–0.05)	5.70 (4.08–7.96)	9.76 (4.02–23.71)

N 7	1 (c) (0.53–1.88)	1 (c) (0.54–1.87)	1 (c) (0.45–2.22)	1 (c) (0.62–1.61)	1 (c) (0.49–2.06)	1 (c) (0.39–2.59)
N 8	1.12 (0.64–1.97)	0.49 (0.29–0.85)	0.80 (0.39–1.67)	0.41 (0.23–0.75)	2.15 (1.36–3.41)	NQ
N 9	0.66 (0.48–0.91)	0.63 (0.45–0.86)	0.49 (0.29–0.83)	0.26 (0.16–0.44)	1.69 (0.84–3.42)	0.51 (0.16–1.63)
TA 7	1.80 (1.34–2.40)	1.15 (0.78–1.69)	0.78 (0.59–1.03)	2.37 (1.79–3.13)	2.42 (1.88–3.12)	1.77 (1.01–3.08)
TA 8	0.98 (0.68–1.40)	0.42 (0.32–0.56)	0.51 (0.37–0.71)	0.87 (0.62–1.22)	0.91 (0.55–1.51)	0.76 (0.50–1.17)
TA 9	1.37 (0.99–1.89)	1.05 (0.81–1.34)	0.79 (0.62–1.00)	0.68 (0.41–1.13)	1.59 (0.76–3.32)	0.15 (0.003–7.94)

NQ, not quantified, detected but only at a very low level; c, calibrator control.

Patients who donated tissue for the research purpose of this study were chronologically numbered. For inter-individual variations, the mRNA transcript levels derived from the first patient number was arbitrarily taken as the calibrator control (*) and set to 1 (for raw data, see Supplementary Information, Table 1S). Quantitative gene expression was carried out exactly as previously described [20,21], with minimum–maximum expression in brackets.  Within each experiment, reactions were performed in triplicate and ‘no-template’ controls were included. Averaged threshold cycle (C_T_) values for each reaction were normalized to *β-ACTIN* values thus giving ΔC_T_ values. Alterations in gene expression were determined by comparison with the tissue value assigned as the calibrator, giving ΔΔC_T_ values. Finally, relative gene expression was calculated using the formula 2^−ΔΔC^_T_.

**Table 3:** Benign prostate tissue (PROS) *vs.* tumour-adjacent (TA) prostate tissue relative gene expression (min-max expression levels).

Patient code	*ERα*	*ERαΔ3*	*ERαΔ5*	*ERβ1*	*ERβ2*	*ERβ5*
PROS 1	1 (c)* (0.71–1.41)	1 (c) (0.25–4.10)	1 (c) (0.06–16.2)	5.95 (5.65–6.27)	0.51 (0.36–0.71)	ND
PROS 2	8.04 (5.59–11.56)	15.6 (8.64–28.17)	1.41 (0.95–2.10)	57.95 (47.1–71.4)	5.04 (3.62–7.03)	ND
PROS 3	0.70 (0.52–0.93)	0.08 (0.02–0.29)	0.56 (0.06–5.63)	1 (c) (0.58–1.72)	1 (c) (0.87–1.15)	1 (c) (0.78–1.29)
TA 13	8.13 (3.15–21.0)	1.07 (0.60–1.91)	12.38 (2.75–55.78)	0.65 (0.54–0.78)	0.74 (0.29–1.9)	0.28 (0.23–0.33)
TA 14	6.59 (3.37–12.88)	1.13 (0.60–2.13)	28.44 (13.79–58.66)	0.54 (0.12–2.49)	1.18 (0.22–6.52)	0.59 (0.11–3.08)
TA 15	10.9 (7.15–16.6)	2.57 (1.61–4.10)	15.4 (8.40–27.27)	1.81 (0.86–3.82)	1.44 (0.77–2.69)	0.78 (0.23–2.61)
PROS 4	1 (c) (0.24–4.25)	1 (c) (0.31–3.22)	1 (c) (0.11–9.14)	Insuff	Insuff	Insuff
PROS 5	0.44 (0.08–2.43)	0.002 (0.001–0.007)	0.63 (0.06–6.76)	Insuff	Insuff	Insuff
PROS 6	11.39 (3.58–36.29)	11.69 (3.72–36.73)	51.4 (6.78–389.6)	Insuff	Insuff	Insuff
TA 16	300.3 (202.4–445.4)	250.2 (180.3–347.1)	24,154.4 (10,085.4–57,849.9)	Insuff	Insuff	Insuff
TA 17	1.26 (0.86–1.86)	0.61 (0.3–1.27)	385.79 (88.89–1,674.4)	Insuff	Insuff	Insuff
TA 18	1.35 (0.46–3.96)	0.81 (0.3–2.21)	28.4 (11.9–68)	Insuff	Insuff	Insuff
PROS 7	1 (c) (0.65–1.54)	1 (c) (0.56–1.8)	1 (c) (0.17–5.91)	4.94 (3.49–6.98)	18.24 (15.1–22.12)	ND
PROS 8	17.39 (13.87–21.8)	37.01 (30.5–44.93)	35.34 (24.23–51.56)	227.5 (207.9–249.0)	95.56 (69.65–131.1)	1.35 (0.31–5.93)
PROS 9	0.48 (0.26–0.89)	0.29 (0.12–0.68)	0.7 (0.05–9.21)	1 (c) (0.79–1.27)	1 (c) (0.54–1.86)	1 (c) (0.64–1.55)
TA 19	6.76 (2.83–16.14)	6.53 (2.79–15.3)	15.31 (2.02–116.23)	10.13 (7.19–14.26)	77.62 (28.89–208.6)	0.02 (0.003–0.21)
TA 20	2.1 (1.83–2.42)	2.07 (1.5–2.86)	10.85 (5.53–21.29)	6.04 (3.58–10.18)	13.01 (6.59–25.68)	1.28 (0.31–5.23)
TA 21	1.67 (1.5–1.86)	1.29 (0.81–2.06)	9.47 (5.43–16.53)	Insuff	Insuff	Insuff
TA 22	Insuff	Insuff	Insuff	1.07 (0.81–1.41)	6.58 (5.18–8.36)	1.41 (0.68–2.93)

ND, not detected; c, calibrator control; Insuff, insufficient material.

Patients who donated tissue for the research purpose of this study were chronologically numbered. For inter–individual variations, the mRNA transcript levels derived from the first patient number was arbitrarily taken as the calibrator control (*) and set to 1 (for raw data, see Supplementary Information, Table 2S). Quantitative gene expression was carried out exactly as previously described [20,21], with minimum–maximum expression in brackets. Within each experiment, reactions were performed in triplicate and ‘no-template’ controls were included. Averaged threshold cycle (C_T_) values for each reaction were normalized to *β-ACTIN* values thus giving ΔC_T_ values. Alterations in gene expression were determined by comparison with the tissue value assigned as the calibrator, giving ΔΔC_T_ values. Finally, relative gene expression was calculated using the formula 2^−ΔΔC^_T_.

**Table 4:** Benign UK prostate tissue (PROS) *vs.* benign Indian prostate tissue (IND) (min–max expression levels).

Patient code	*ERα*	*ERαΔ3*	*ERαΔ5*	*ERβ1*	*ERβ2*	*ERβ5*
PROS 10	1 (c)* (0.2–4.93)	1 (c) (0.15–6.88)	1 (c) (0.57–1.74)	1 (c) (0.74–1.36)	1 (c) (0.48–2.09)	1 (c) (0.47–2.13)
PROS 11	1.86 (1.31–2.64)	0.45 (0.22–0.95)	0.3 (0.15–0.59)	0.54 (0.39–0.74)	0.33 (0.2–0.55)	8.16 (2.0–33.22)
PROS 12	1.40 (0.87–2.24)	0.34 (0.26–0.44)	0.35 (0.26–0.48)	0.21 (0.1–0.42)	0.26 (0.13–0.55)	1.22 (0.17–8.8)
IND 11	Insuff	Insuff	Insuff	0.58 (0.21–1.65)	0.42 (0.22–0.8)	0.23 (0.01–6.1)
IND 12	0.82 (0.58–1.18)	0.07 (0.02–0.3)	0.26 (0.2–0.34)	0.04 (0.02–0.06)	0.07 (0.05–0.1)	0.02 (0.01–0.03)
IND 13	Insuff	Insuff	Insuff	65.12 (11.2–375.4)	0.99 (0.16–6.24)	6.12 (0.98–38.19)
IND 14	0.79 (0.61–1.04)	0.16 (0.1–0.26)	0.03 (0.02–0.05)	Insuff	Insuff	Insuff
IND 15	5.54 (3.53–8.69)	4.23 (2.91–6.16)	1.58 (0.19–12.85)	Insuff	Insuff	Insuff

PROS 7	1 (c) (0.63–1.59)	1 (c) (0.78–1.28)	1 (c) (0.60–1.67)	1 (c) (0.53–1.88)	1 (c) (0.35–2.83)	1 (c) (0.45–2.23)
PROS 8	36.59 (31.22–42.88)	19.12 (16.01–22.82)	142.7 (86.53–235.3)	29.24 (19.35–44.2)	78.43 (21.56–285.3)	654.8 (61.56–6,966.2)
PROS 9	0.48 (0.31–0.74)	0.18 (0.13–0.25)	5.74 (1.11–29.65)	0.41 (0.25–0.67)	0.04 (0.02–0.09)	2,341.7(1,122.7–4,884.3)
IND 1	0.44 (0.31–0.62)	0.03 (0.01–0.09)	1.19 (0.14–10.4)	0.005 (0.004–0.007)	0.001 (0–0.011)	0.004 (0.003–0.005)
IND 2	5.27 (2.79–9.95)	3.09 (1.81–5.26)	ND	3.0 (2.16–4.16)	2.69 (0.47–15.37)	3.07 (0.57–16.45)
IND 3	1.53 (1.22–1.92)	1.11 (0.62–1.99)	3.23 (1.61–6.5)	1.33 (0.81–2.16)	0.18 (0.004–8.7)	0.29 (0.01–6.63)

PROS 1	Insuff	Insuff	Insuff	1 (c) (0.6–1.67)	1 (c) (0.1–10.14)	1 (c) (0.19–5.37)
PROS 2	Insuff	Insuff	Insuff	8.46 (7.15–10.01)	23.5 (21.1–26.17)	0.45 (0.1–2.04)
PROS 3	Insuff	Insuff	Insuff	0.12 (0.08–0.17)	1.96 (1.4–2.75)	0.16 (0.02–1.71)
IND 4	Insuff	Insuff	Insuff	0.23 (0.1–0.54)	4.79 (3.62–6.34)	0.09 (0.06–0.13)
IND 5	Insuff	Insuff	Insuff	ND	0.25 (0.03–2.24)	8.61 (2.59–28.69)
IND 6	Insuff	Insuff	Insuff	20.02 (15.34–26.12)	9.47 (5.66–15.85)	4.37 (2.59–7.35)

PROS 4	Insuff	Insuff	Insuff	1 (c) (0.67–1.49)	1 (c) (0.43–2.33)	1 (c) (0.19–5.32)
PROS 5	Insuff	Insuff	Insuff	0.04 (0.02–0.08)	0.13 (0.09–0.17)	0.02 (0.02–0.02)
PROS 6	Insuff	Insuff	Insuff	10.15 (4.1–25.11)	2.87 (1.31–6.26)	35.7 (15.2–83.8)
IND 7	Insuff	Insuff	Insuff	13.64 (10.48–17.77)	0.99 (0.66–1.49)	6.26 (1.06–37.0)
IND 8	Insuff	Insuff	Insuff	0.02 (0.02–0.03)	0.2 (0.14–0.29)	2.26 (1.31–3.89)
IND 10	Insuff	Insuff	Insuff	0.04 (0.01–0.13)	0.33 (0.19–0.56)	0.01 (0.002–0.03)

ND, not detected; c, calibrator control; Insuff, insufficient material.

Patients who donated tissue for the research purpose of this study were chronologically numbered. For inter–individual variations, the mRNA transcript levels derived from the first patient number was arbitrarily taken as the calibrator (*) and set to 1 (for raw data, see Supplementary Information, Table 3S). Quantitative gene expression was carried out exactly as previously described [20,21], with minimum–maximum expression in brackets. Within each experiment, reactions were performed in triplicate and ‘no–template’ controls were included. Averaged threshold cycle (C_T_) values for each reaction were normalized to *β-ACTIN* values thus giving ΔC_T_ values. Alterations in gene expression were determined by comparison with the tissue value assigned as the calibrator, giving ΔΔC_T_ values. Finally, relative gene expression was calculated using the formula 2^−ΔΔC^_T_.

## Appendix

**Table 1S:** Benign *vs.* tumour-adjacent endometrial tissue, mean C_T_ values (corresponding *β-ACTIN* C_T_ value).

Code	*ERα*	*ERαΔ3*	*ERαΔ5*	*ERβ1*	*ERβ2*	*ERβ5*
N1	25.0 (19.2)	30.6 (19.2)	31.2 (19.2)	33.9 (18.7)	33.4 (18.7)	37.0 (18.7)
N2	24.0 (19.2)	30.0 (19.2)	31.5 (19.2)	32.8 (18.5)	32.6 (18.5)	35.1 (18.5)
N3	23.9 (19.4)	28.6 (19.4)	30.2 (19.4)	34.1 (18.8)	33.4 (18.8)	36.9 (18.8)
TA1	24.7 (19.0)	30.4 (19.0)	31.9 (19.0)	31.1 (18.7)	32.9 (18.7)	33.9 (18.7)
TA2	23.0 (17.7)	28.3 (17.7)	30.2 (17.7)	32.4 (17.7)	30.9 (17.7)	34.6 (17.7)
TA3	24.4 (18.8)	30.1 (18.8)	31.2 (18.8)	34.0 (18.8)	32.7 (18.8)	37.1 (18.8)

N4	26.4 (20.8)	31.1 (20.8)	31.5 (20.8)	30.1 (19.4)	33.5 (19.4)	39.4 (19.4)
N5	29.0 (22.8)	32.5 (22.8)	33.7 (22.8)	20.7 (21.3)	34.5 (21.3)	40.3 (21.3)
N6	26.0 (20.3)	30.8 (20.3)	30.8 (20.3)	34.1 (19.0)	33.7 (19.0)	37.7 (19.0)
TA4	24.6 (20.2)	29.6 (20.2)	29.5 (20.2)	24.7 (18.3)	32.9 (18.3)	34.3 (18.3)
TA5	24.3 (20.3)	29.5 (20.3)	29.8 (20.3)	35.3 (18.6)	33.4 (18.6)	37.0 (18.6)
TA6	27.2 (22.7)	32.2 (22.7)	33.3 (22.7)	37.2 (21.4)	33.0 (21.4)	38.2 (21.4)

N7	24.5 (19.0)	29.4 (19.0)	30.5 (19.0)	33.1 (18.9)	33.5 (18.9)	37.2 (18.9)
N8	24.4 (19.1)	30.6 (19.1)	30.9 (19.1)	34.2 (18.8)	32.3 (18.8)	58.7 (18.8)
N9	26.0 (19.9)	31.0 (19.9)	32.4 (19.9)	36.1 (20.0)	33.8 (20.0)	39.2 (20.0)
TA7	24.0 (19.4)	29.7 (19.4)	31.3 (19.4)	32.1 (19.1)	32.5 (19.1)	36.6 (19.1)
TA8	24.1 (18.5)	30.2 (18.5)	31.0 (18.5)	32.8 (18.5)	33.2 (18.5)	37.2 (18.5)
TA9	23.2 (18.2)	28.6 (18.2)	30.0 (18.2)	32.6 (17.9)	31.8 (17.9)	38.9 (17.9)

N, benign endometrial tissue code; TA, tumour-adjacent endometrial tissue code.

This table presents the raw data required for the relative gene expression analysis shown in Table 2. The mean C_T_ values of each gene investigated and the mean C_T_ value of *β-ACTIN* (in brackets), is given for each patient. Gene expression analysis is performed by comparing *β-ACTIN* values with those of the gene of interest, relative to one ‘control’ patient known as the calibrator (consequently assigned a gene expression value of 1) in order to determine relative inter-patient differences (see Table 2).

**Table 2S:** Benign *vs.* tumour-adjacent prostate, mean C_T_ values (corresponding *β-ACTIN* C_T_ value).

Code	*ERα*	*ERαΔ3*	*ERαΔ5*	*ERβ1*	*ERβ2*	*ERβ5*
PROS 1	36.4 (23.9)	39.5 (23.9)	43.9 (23.9)	37.0 (24.4)	37.2 (24.4)	57.1 (24.4)
PROS 2	34.4 (24.9)	36.6 (24.9)	44.4 (24.9)	34.8 (23.3)	35.0 (23.3)	53.2 (23.3)
PROS 3	33.8 (20.7)	40.1 (20.7)	41.6 (20.7)	36.5 (20.3)	33.1 (20.3)	37.5 (20.3)
TA 13	29.1 (19.7)	35.2 (19.7)	36.0 (19.7)	36.3 (19.5)	32.8 (19.5)	38.6 (19.5)
TA 14	28.8 (19.0)	34.5 (19.0)	34.2 (19.0)	35.4 (18.3)	31.0 (18.3)	36.4 (18.3)
TA 15	30.0 (21.0)	35.3 (21.0)	37.1 (21.0)	36.5 (21.2)	33.5 (21.2)	38.8 (21.2)

PROS 4	41.3 (29.8)	43.0 (29.8)	56.1 (29.8)	Insuff	Insuff	Insuff
PROS 5	39.0 (26.3)	48.2 (26.3)	53.2 (26.3)	Insuff	Insuff	Insuff
PROS 6	37.7 (29.7)	39.3 (29.7)	50.3 (29.7)	Insuff	Insuff	Insuff
TA 16	32.6 (29.4)	34.6 (29.4)	41.1 (29.4)	Insuff	Insuff	Insuff
TA 17	37.3 (26.1)	40.0 (26.1)	43.8 (26.1)	Insuff	Insuff	Insuff
TA 18	37.7 (26.6)	40.1 (26.6)	48.1 (26.6)	Insuff	Insuff	Insuff

PROS 7	36.2 (25.8)	38.6 (25.8)	44.7 (25.8)	37.2 (25.7)	34.6 (25.7)	56.0 (25.7)
PROS 8	31.6 (25.3)	32.9 (25.3)	39.1 (25.3)	31.2 (25.2)	31.7 (25.2)	42.1 (25.2)
PROS 9	33.4 (21.9)	36.5 (21.9)	41.3 (21.9)	35.0 (21.2)	34.2 (21.2)	38.5 (21.2)
TA14 19	33.0 (25.3)	35.5 (25.3)	40.4 (25.3)	36.0 (25.5)	23.3 (25.5)	48.2 (25.5)
TA17 20	31.0 (21.7)	33.5 (21.7)	37.2 (21.7)	32.6 (21.5)	30.8 (21.5)	38.4 (21.5)
TA21 21	32.1 (22.4)	34.9 (22.4)	38.2 (22.4)	Insuff	Insuff	Insuff
TA8 22	Insuff	Insuff	Insuff	33.8 (20.1)	30.4 (20.1)	36.8 (20.1)

PROS, benign prostate tissue code; TA, tumour-adjacent prostate tissue code; Insuff, insufficient material.

This table presents the raw data required for the relative gene expression analysis shown in Table 3. The mean C_T_ values of each gene investigated and the mean C_T_ value of *β-ACTIN* (in brackets), is given for each patient. Gene expression analysis is performed by comparing *β-ACTIN* values with those of the gene of interest, relative to one ‘control’ patient known as the calibrator (consequently assigned a gene expression value of 1) in order to determine relative inter–patient differences (see Table 3).

**Table 3S:** UK *vs.* India prostate, mean C_T_ values (corresponding *β-ACTIN* C_T_ value).

Code	*ERα*	*ERαΔ3*	*ERαΔ5*	*ERβ1*	*ERβ2*	*ERβ5*
PROS 10	39.2 (26.9)	40.2 (26.9)	41.5 (26.9)	39.8 (25.7)	35.8 (25.7)	45.8 (25.7)
PROS 11	36.1 (24.7)	39.1 (24.7)	41.1 (24.7)	38.4 (23.4)	35.1 (23.4)	40.6 (23.4)
PROS 12	33.9 (22.2)	37.0 (22.2)	38.2 (22.2)	37.5 (21.1)	33.2 (21.1)	41.0 (21.1)
IND11	Insuff	Insuff	Insuff	37.8 (22.9)	34.3 (22.9)	45.2 (22.9)
IND12	34.2 (21.8)	38.8 (21.8)	38.3 (21.8)	40.4 (21.6)	35.6 (21.6)	47.7 (21.6)
IND13	Insuff	Insuff	Insuff	33.8 (25.7)	35.8 (25.7)	43.3 (25.7)
IND14	34.6 (22.0)	37.9 (22.0)	41.6 (22.0)	Insuff	Insuff	Insuff
IND15	36.8 (27.0)	38.2 (27.0)	40.9 (27.0)	Insuff	Insuff	Insuff

PROS 7	35.7 (26.2)	37.3 (26.2)	45.4 (26.2)	36.2 (26.1)	48.7 (26.1)	51.4 (26.1)
PROS 8	30.2 (25.9)	32.8 (25.9)	38.0 (25.9)	30.8 (25.6)	41.9 (25.6)	41.6 (25.6)
PROS 9	32.9 (22.3)	35.9 (22.3)	39.0 (22.3)	34.0 (22.6)	50.0 (22.6)	36.7 (22.6)
IND1	33.7 (23.1)	39.1 (23.1)	42.0 (23.1)	39.6 (21.8)	53.9 (21.8)	55.1 (21.8)
IND2	35.7 (28.6)	38.1 (28.6)	42.4 (28.6)	35.4 (26.9)	48.1 (26.9)	50.6 (26.9)
IND3	33.7 (24.9)	35.9 (24.9)	ND	33.0 (23.4)	48.5 (23.4)	50.5 (23.4)
PROS 1	Insuff	Insuff	Insuff	36.6 (23.8)	37.8 (23.8)	41.3 (23.8)

PROS 2	Insuff	Insuff	Insuff	34.4 (24.6)	34.1 (24.6)	43.2 (24.6)
PROS 3	Insuff	Insuff	Insuff	36.5 (20.5)	33.6 (20.5)	40.6 (20.5)
IND4	Insuff	Insuff	Insuff	40.3 (23.4)	37.1 (23.4)	46.2 (23.4)
IND5	Insuff	Insuff	Insuff	ND	46.0 (30.0)	44.4 (30.0)
IND6	Insuff	Insuff	Insuff	32.1 (23.6)	34.4 (23.6)	38.9 (23.6)

PROS 4	Insuff	Insuff	Insuff	38.1 (27.3)	37.3 (27.3)	44.4 (27.3)
PROS 5	Insuff	Insuff	Insuff	38.7 (23.2)	36.2 (23.2)	45.9 (23.2)
PROS 6	Insuff	Insuff	Insuff	35.3 (27.8)	36.4 (27.8)	39.8 (27.8)
IND7	Insuff	Insuff	Insuff	32.4 (25.3)	35.4 (25.3)	39.8 (25.3)
IND8	Insuff	Insuff	Insuff	37.2 (21.0)	33.4 (21.0)	37.0 (21.0)
IND10	Insuff	Insuff	Insuff	38.8 (23.4)	35.0 (23.4)	47.6 (23.4)

ND, not detected; Insuff, insufficient material.

PROS, Benign UK-resident prostate tissue code; IND, Benign India-resident prostate tissue code

This table presents the raw data required for the relative gene expression analysis shown in Table 4. The mean C_T_ values of each gene investigated and the mean C_T_ value of *β-ACTIN* (in brackets), is given for each patient. Gene expression analysis is performed by comparing *β-ACTIN* values with those of the gene of interest, relative to one ‘control’ patient known as the calibrator (consequently assigned a gene expression value of 1) in order to determine relative inter–patient differences (see Table 4).

**Table 4S:** Prostate samples demographic details.

Code	Age (y)	PSA (ng/mL)	Gleason grade
PROS 1	71	3.75	NA
PROS 2	81	4.99	NA
PROS 3	72	NK	NA
PROS 4	72	4.96	NA
PROS 5	73	2.49	NA
PROS 6	74	6.25	NA
PROS 7	81	5	NA
PROS 8	62	5.7	NA
PROS 9 (open)	82	34	NA
PROS 10	79	3.75	NA
PROS 11	71	4.19	NA
PROS 12 (open)	73	5	NA

TA 13	60	11.5	3 + 3
TA 14	61	8.2	3 + 3
TA 15	64	8.7	4 + 4
TA 16	65	6.5	3 + 3
TA 17	56	2.7	benign
TA 18	67	NK	benign
TA 19	NK	NK	NK
TA 20	66	9	3 + 3
TA 21	57	5.3	3 + 3
TA 22	63	5.8	3 + 4
IND 1	58	1.2	NA
IND 2	60	2.4	NA
IND 3	63	0.3	NA
IND 4	76	3.2	NA
IND 5	75	0.3	NA
IND 6	65	0.2	NA
IND 7	79	2.5	NA
IND 8	74	0.3	NA
IND 9	65	0.6	NA
IND 10	NK	NK	NA
IND 11	62	3.3	NA
IND 12	47	2.4	NA
IND 13	64	2.4	NA
IND 14	60	0.3	NA
IND 15	74	1.4	NA

NK, not known; NA, not applicable; open, open prostatectomy.

PROS, Benign UK-resident prostate tissue code; TA, tumour-adjacent prostate tissue code; IND, Benign India-resident prostate tissue code.

**Table 5S:** Endometrial samples demographic details.

Code	Age (y)	Histology	Stage
N1	39	proliferative	NA
N2	46	early proliferative	NA
N3	42	proliferative	NA
N4	39	proliferative	NA
N5	46	proliferative	NA
N6	51	proliferative	NA
N7	42	proliferative	NA
N8	43	proliferative to early secretory with simple hyperplasia	NA
N9	43	proliferative	NA

TA1	58	G2 endometrioid	3a
TA2	74	G2 endometrioid	1c
TA3	67	G2 endometrioid	1b
TA4	84	G2 endometrioid	1a
TA5	57	G2 endometrioid	1a
TA6	77	G2 endometrioid	1c
TA7	70	G2 endometrioid	1b
TA8	62	G2 endometrioid	1b
TA9	62	G2 endometrioid	2a

N, benign endometrial tissue code;

TA, tumour-adjacent endometrial tissue code;

NA, not applicable.

## Abbreviations

C: calibrator control
CaP: prostate cancer
C_T_: threshold cycle
ER: oestrogen receptor
GSPBS: 5% normal goat serum in PBS
PI: propidium iodide
PMT: photomultiplier tubes
PROS: benign prostate tissues
PSA: prostate-specific antigen
RRP: radical retro-pubic prostatectomy
RT-PCR: real time polymerase chain reaction
TA: tumour-adjacent
TURP: trans-urethral resection of the prostate
